# Sample Entropy of the Heart Rate Reflects Properties of the System Organization of Behaviour

**DOI:** 10.3390/e20060449

**Published:** 2018-06-08

**Authors:** Anastasiia V. Bakhchina, Karina R. Arutyunova, Alexey A. Sozinov, Alexander V. Demidovsky, Yurii I. Alexandrov

**Affiliations:** 1 Institute of Psychology of Russian Academy of Sciences, Laboratory of Neural Bases of Mind Named after V.B. Shvyrkov, 129366 Moscow, Russia; 2 Department of Psychophysiology, National Research University Nizhny Novgorod State University Named after N.I. Lobachevsky, 603950 Nizhny Novgorod, Russia; 3 Computer Science Department, National Research University Higher School of Economics, 603014 Nizhny Novgorod, Russia; ademidovskij@hse.ru or; 4 Department of Psychology, National Research University Higher School of Economics, 101000 Moscow, Russia

**Keywords:** heart rate variability, irregularity, sample entropy, functional systems, individual development, stress, alcohol administration, learning

## Abstract

Cardiac activity is involved in the processes of organization of goal-directed behaviour. Each behavioural act is aimed at achieving an adaptive outcome and it is subserved by the actualization of functional systems consisting of elements distributed across the brain and the rest of the body. This paper proposes a system-evolutionary view on the activity of the heart and its variability. We have compared the irregularity of the heart rate, as measured by sample entropy (SampEn), in behaviours that are subserved by functional systems formed at different stages of individual development, which implement organism-environment interactions with different degrees of differentiation. The results have shown that SampEn of the heart rate was higher during performing tasks that included later acquired knowledge (foreign language vs. native language; mathematical vocabulary vs. general vocabulary) and decreased in the stress and alcohol conditions, as well as at the beginning of learning. These results are in line with the hypothesis that irregularity of the heart rate reflects the properties of a set of functional systems subserving current behaviour, with higher irregularity corresponding to later acquired and more complex behaviour.

## 1. Introduction

Studies of the physiological bases of behaviour do not often take into account the processes that occur outside the anatomical borders of the brain. The mental processes, such as perceptions, thoughts, and feelings, for a long time have been viewed without considering the physiological state of the body. At the current stage of the development of cognitive sciences more attention is paid to the analyses of “embodied cognition” and whole-organism integration that underpin behaviour and psychological processes [1,2,3,4]; however, the mechanisms of such integration are still a debatable question.

From the physiological perspective, the interest in the “mind-body” relationship is becoming obvious as the number of studies on autonomic regulation of processes involving internal organs is increasing [5,6]. Some previous studies were focused on how different subcortical structures modulate the activity of internal organs but now more attention is paid to the corticovisceral coordination [7,8,9]. In general, it appears important to develop an integrated approach to study the psychophysiological bases of behaviour organization that would take into account the significance of the impact of the body’s physiological states. In this direction, the phenomenon of heart rate variability is of interest.

The heart beats with complicated oscillations in coherence with the functioning of the entire body. Heart rate variability (HRV) denotes the change in the time intervals between adjacent heartbeats and it is necessary to regulate the transport of resources through the body in order to adapt to external challenges and achieve optimal performance [10]. Therefore, the cardiovascular activity, and particularly, heart rate, are viewed in psychophysiological studies as related to relevant behavioural processes.

From the physiological perspective, the primary origin of HRV is related to the activity of the sympathetic and parasympathetic subdivisions of the autonomic nervous system (ANS) [11]. Higher HRV at rest supports a better performance at a number of tasks [12]. Several theories [13] explain why large HRV is associated with physical fitness and youth, while physical and psychological stress is associated with decreased HRV. However, such purely physiological models are limited in explaining the HRV related to behaviour. Moreover, although it is clear how the activity of ANS is reflected in HRV, the regulation of heart rate by cortical structures is still an unanswered question [14,15,16]. Here, we address this problem from the positions of the system-evolutionary theory and propose a model explaining HRV in relation to neuronal processes in the brain.

The system-evolutionary theory [3,4,17] was built upon the fundamentals of P.K. Anokhin’s theory of functional systems [18], which suggests that morphologically different components of the body and brain comprise functional systems, in which their co-operative activity leads to achieving adaptive results within the organism-environment relations. P.K. Anokhin emphasized that breathing and blood flow observed during performing different behavioural acts may seem the same, but they are actually different, because their characteristics depend on the behavioural result that is being achieved by means of performing this particular act [19]. The system-evolutionary theory considerably extends this approach and proposes to view neurons and other body cells, as “organisms within the organism”, i.e., the activity of each cell is aimed at satisfying its metabolic needs by means of interacting with the environment and other cells [3]. Neurons and body cells joint cooperative activity leads to the adaptive result that is a new relation between an organism and the environment. Each novel way of adaptive organism-environment interaction underlies the formation of a new functional system represented by the cells that were active when achieving this adaptive outcome. The subsequent actualization of this functional system underlies the realization of this behavioural act, which can be considered as an element of subjective experience. Thus, a functional system is understood as a dynamic organization of activity of components across different anatomical localizations, both in the brain and the rest of the body, which provides the achievement of an adaptive result for the whole organism.

When considered within the framework of the system-evolutionary theory, HRV originates in cooperation of the heart with the other components of actualized functional systems, including neuronal groups. This approach suggests that HRV depends on the behavioural results that were achieved during the organism-environment interactions and, therefore, may differ between sequential behavioural acts. The current study is aimed at analyzing and examining this dependency and its implications.

It has been shown [2,3,20] that a newly formed complex instrumental behaviour is based on the simultaneous activation of the corresponding “new” functional system with the older systems that had been formed at previous stages of individual development. The older systems become involved in many behavioural patterns as they belong to the elements of individual experience that are common for various acts. Therefore, the system organization of behaviour represents the history of behavioural development. Multiple systems are involved in behaviour, and each of them was formed at a certain stage of development of the current behaviour [21].

The structure of experience becomes more complex and differentiated during individual development. The formation of new functional systems results in growing complexity and the degree of differentiation of organism-environment relations. A new functional system does not replace previously formed systems, but instead, it is “superimposed” on them [20]. Individual development can be considered as the process of increasing differentiation along with the number of learned behaviors [22,23]. Therefore, behaviour formed at later stages of individual development provides a more differentiated (more detailed and accurate) organism-environment relation and supported by a larger set of systems and links between them. From this view, heart activity depends on the system characteristics of current behaviour and HRV is hypothesized to be higher when an individual performs recently formed behaviour than behaviour formed at earlier stages of development. This is the first hypothesis that is explored in our study.

Psychological and physiological stress along with other factors, such as alcohol intoxication, lead to a temporary increase of the role of earlier formed systems in the organization of behaviour [24,25]. This phenomenon of “system dedifferentiation” is usually accompanied by increased emotional arousal, decreased cognitive control, less detailed perception and performance, preference of intuitive strategies in decision making to rational ones, etc. On the neuronal level, it is shown that alcohol decreases the number of activated cortical neurons specialized in relation to later formed systems [26]. This implies that HRV would decrease in the context of system dedifferentiation, which is the second hypothesis that is explored in this study.

Thus, the goal of the study was to examine the interrelations between the systems supporting behaviour and the HRV during the performance of this behaviour. We hypothesized that a lower HRV is observed during performing early-formed behaviour in comparison with behaviour formed more recently, and HRV decreases in the contexts of system dedifferentiation.

## 2. Materials and Methods

### 2.1. Ethical Statement

All of the participants gave written informed consent to take part in the study after receiving an explanation of the procedures.

The study was conducted in accordance with the Declaration of Helsinki. The Ethics Committee of the Federal State-Financed Institution, Institute of Psychology, and Russian Academy of Sciences (Moscow) approved the experimental protocols and the specific consent procedure used in this study, and assessed it as safe for the participants’ psychic and physical health. All of the participants were native Russian-speakers and were paid for their participation.

### 2.2. Heart Rate Measurement & Heart Rate Variability Analyses

In all of the experiments, RR-intervals (the time periods between consecutive heartbeats) were measured using a miniature ECG sensor (HxM; sampling rate 250 Hz, Zephyr Technology, Annapolis, MD, USA, www.zephyranywhere.com). Participants wore a special chest belt with two plastic electrodes that were located in the first and second chest leads. Batch data transmission from the sensor to a mobile device was carried out through the wireless protocol Bluetooth. Connecting, data transmission and storage on the mobile device were performed using custom software “HR-Reader” [developed by Kozhevnikov V.V.] for Android OS.

Acquired sequences of RR-intervals were pre-processed before proceeding to the analysis. The sequences with abnormal beats and any artifacts (ectopic beats, coughs, and motion artifacts) were excluded from the analyses. The artifacts were identified as RR-intervals that did not satisfy the condition │RR_i_-RR_i−1_│ < 0.7 × (RR_i_-RR_i−1_)/2. Thus, we only analyzed sequences that were free from artifacts.

For estimation of heart rate irregularity we used the sample entropy (SampEn) as a set of measures of system irregularity reporting on similarity in time series. SampEn can be applied to relatively short and noisy data; it is largely independent of record length and displays relative consistency under circumstances. SampEn (m, r, N) is precisely the negative natural logarithm of the conditional probability that two vectors that are similar for m points remain similar at the next point, where self-matches are not included in calculating the probability (1) [27].

(1) $$\mathrm{SampEn}\left(m,\;r,\;N\right)=-\ln\frac{A}{B},$$

The parameter N is the length of the time series, m is the length of vectors to be compared, and r is the tolerance for accepting matches. A is the number of pairs of vectors (x) for m points that satisfy the condition d[xm(i), xm(j)] ≤ r, and B is the number of pairs of vectors (x) for (m+1) points that satisfy the condition d[xm(i), xm(j)] ≤ r. Thus, a low value of SampEn reflects a high degree of regularity. The parameters m and r were fixed: m = 2, r = 0.5 × SDNN (SDNN—standard deviation of RR-intervals). It had been shown previously that higher values of r were accompanied by lower dispersion of SampEn [27]. While a lot of studies use r = 0.2 × SDNN, we chose to use a larger value of r to avoid extra dispersion in our small samples. It is important to note that SampEn still reliably distinguishes the processes with different degrees of order when calculated with r = 0.5 × SDNN [27]. Calculations were made using the cross-platform software that contained a set of algorithms implemented in Python and PyQt5 as the UI rendering framework. The source code is available in the open source repo (https://github.com/demid5111/approximate-enthropy) under the MIT license.

Additionally the time domain indexes of HRV (mean (av-RR, ms) and standard deviation (SDNN, ms) of RR-intervals) were calculated.

It is accepted that irregularity of heart rate (measured by SampEn) and its variability (measured by SDNN) can correlate, but these correlations are not linear [28]. Therefore, we can face such modes of heart activity which differ in irregularity and have the same variability, and vice versa. For example, SDNN, as an index of HRV, is modulated by breathing, while SampEn is independent of the characteristics of respiratory arrhythmia. Thus, SampEn and SDNN are both HRV indexes partly complementing each other. In this work, we used SampEn as an index of irregularity, SDNN as an index of variability, and av-RR as an index of frequency of the heart rate.

### 2.3. Experimental Protocols

The study included five experiments. In the beginning of each experiment, the participants filled questionnaires about their demographics, health, and well-being during the past several days prior to the experiment. Then, the participants were informed about the details of experimental procedures.

#### 2.3.1. Experiment 1

**Figure 1:**
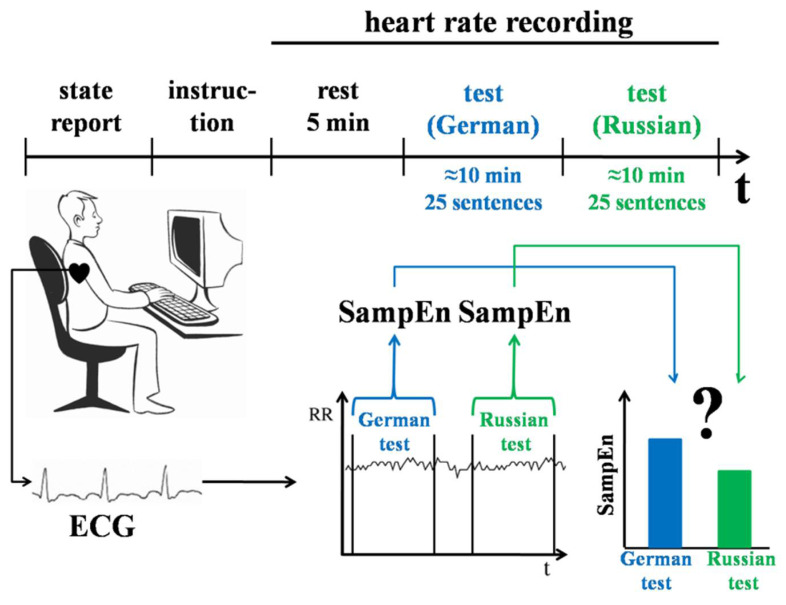
Design of Experiment 1. Participants performed two computer tests: one in a foreign language (German) and the other one in a native language (Russian). Participants’ heart rate was recorded. Sample entropy (SampEn), mean (av-RR), and standard deviation (SDNN) values were calculated for RR-intervals and compared between the periods of performing the German and Russian tests.

The aim of Experiment 1 was to compare HRV during the realization of behaviour formed at earlier stages of individual development with behaviour formed later in life (see Figure 1). Participants performed a set of linguistic tasks either in native language (earlier formed), or foreign language (later formed). It had been shown previously that foreign-language processing reduces the impact of intuition and/or increases the impact of deliberation on individuals’ choices [29]. Using a foreign language affects moral judgment by blunting emotional reactions that are associated with violation of moral rules [30]. It has been shown in EEG studies that the latencies of event-related potentials are longer when performing tasks in foreign language as compared to performing them in native language, which has been viewed as an indicator of different degrees of automaticity and quantity of the subprocesses that are involved in sentence comprehension [31]. Thus, solving tasks using a foreign language can be considered as a later formed behaviour based on the activation of a wider distributed neuronal subserving in the brain, as opposed to solving tasks using a native language [32].

Participants (N = 29, 25 females, 18 to 26 years old, mean = 20, median = 20) were native Russian speakers recruited at the Nizhny Novgorod Linguistic State University, named after N.A. Dobrolubov, where they were studying German as a foreign language. For some participants (N = 11), German was a primary foreign language and they spent 6–18 (mean = 12.2; median = 13) years studying it, while for others (N = 18) it was a secondary foreign language which they studied for 1–5 years (mean = 2.6; median = 2). We compared the number of mistakes, response times (ms), and heart rate indexes during performing the tests in these two groups of participants. The number of mistakes in the German-language test was lower in the group who had been studying German as a primary foreign language for a longer period of time (6–18 years of studying: Med = 5, Q1 = 2, Q3 = 10; 1–5 years of studying: Med = 12, Q1 = 6, Q3 = 13; U = 50, Z = 2.17, *p* < 0.03, Mann-Whitney test). Therefore, we analyzed the data for these two groups separately.

The participants performed two tests: one in German and one in Russian. The order of the tests was counterbalanced. Both tests consisted of 25 sentences that were presented on a computer screen one at a time in a randomized order. Each sentence had a missing word, which was always a noun. The task was to complete the sentences. The time to perform the task was not limited.

Each participant was seated in a quiet room approximately 50 cm from a computer screen. Sentences were presented in white letters against a black background in the center of the screen. A standard computer keyboard was used. To type in a missing word, participants had to press the spacebar and then press the appropriate keys on the keyboard, followed by pressing the Enter key to proceed to the next sentence. The tests were presented using custom experiment software. The cross-platform software was implemented in Java to support conducting experiments with wide configuration capabilities. The source code is available in the open source repo (https://bitbucket.org/ademidovskij/test_me_words) under the MIT license.

Participants’ test performance, response times (in ms), and heart rate were recorded. Using Wilcoxon test, we compared SampEn, av-RR, and SDNN values calculated for RR-intervals during the periods of performing the language tests in German and Russian.

#### 2.3.2. Experiment 2

**Figure 2:**
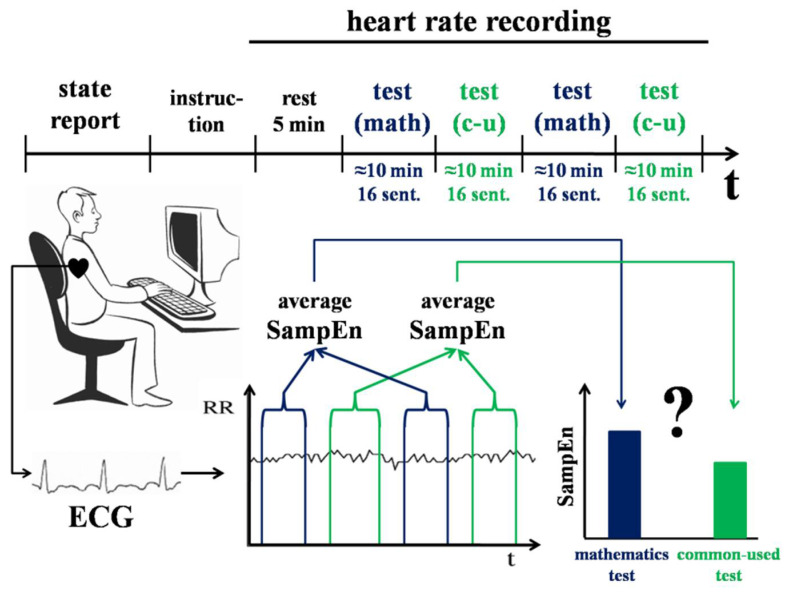
Design of Experiment 2. Participants were asked to complete sentences with missing words. Two groups of sentences included words with different age of acquisition: early acquired commonly used words (“c-u”) and later acquired mathematical terms (“math”). Participants’ heart rate was recorded while performing the task. SampEn, av-RR, and SDNN values were calculated for RR-intervals and compared between the periods of using earlier and later acquired words.

The aim of Experiment 2 was to compare HRV during behaviour that was formed at earlier or later stages of individual development (see Figure 2). As opposed to Experiment 1, in this case, we used a linguistic task presented in the participants’ native language (Russian) to avoid inter-lingual differences. The task included words that were typically learnt at a different age [33]. It has been shown that early-acquired words are recalled, read, and recognized faster than later acquired words [34]. Early-acquired adjectives are also rated by participants as more emotional [22]. Therefore, we considered using early-acquired words as related to earlier formed and less detailed behaviour.

Participants (N = 35, 5 females, 23 to 37 years old, mean = 27.78, median = 28) were professional mathematicians with work experience of 1–10 years (median = 4.84 years). They were presented with two types of tests. The first test consisted of 32 sentences, which contained mathematical terms. These terms are usually learned at an undergraduate level (age of acquisition is 18–19 years old). For example, “A normal is a vector that is perpendicular to a given object”. The second test consisted of 32 sentences, which contained commonly used words that are familiar from childhood (age of acquisition is 5–6 years old). For example, “Plasticine is a material for modelling figures”. Sentences from the two tests had equal linguistic characteristics, such as the number of words, syllables, letters and Fog’s index (a value of text’s complexity). Each sentence had a missing word and the experimental task was to complete these sentences. The time to perform the task was not limited. The task was organized in four sets, 16 items each: two of them contained mathematical terms and the two others contained commonly used words. The presentation of the sets was counterbalanced. The sentences in each set were mixed and presented one at a time in a randomized order.

Each participant was seated in a quiet room approximately 50 cm from a computer screen. Sentences were presented in white letters against a black background in the center of the screen. An underscored gap indicated a place for a missing word. A standard computer keyboard was used. To type a missing word, the participants had to press the spacebar and then press the appropriate keys on the keyboard, followed by pressing the Enter key to proceed to the next sentence. The tests were presented using custom experiment software. The cross-platform software was implemented in Java to support conducting experiments with wide configuration capabilities. The source code is available in the open source repo (https://bitbucket.org/ademidovskij/test_me_words) under the MIT license.

Participants’ test performance, response times (in ms), and heart rate were recorded. SampEn, av-RR, and SDNN values were calculated for RR-intervals during performing both sets of sentences. We averaged SampEn, av-RR, and SDNN values for the sentences containing mathematical terms and the sentences containing commonly used words, and then these averaged SampEn, av-RR, and SDNN values were compared using Wilcoxon test.

#### 2.3.3. Experiment 3

**Figure 3:**
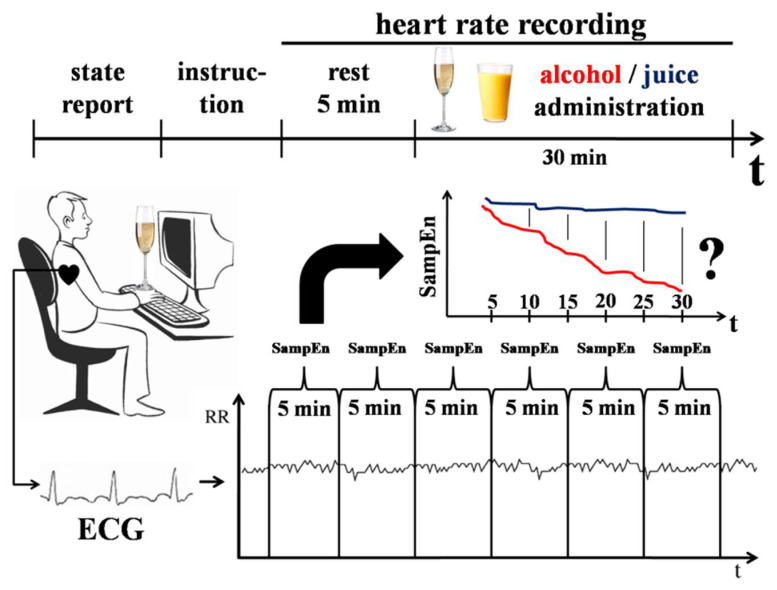
Design of Experiment 3. Heart rate was recorded during a 30 min period while participants were drinking a beverage and watching a video. SampEn was calculated for sequential 5 min sections of RR-intervals. The dynamics of SampEn, av-RR, and SDNN values in the control (juice + water) and experimental (juice + alcohol) conditions were compared.

The aim of Experiment 3 was to study the dynamics of HRV in the conditions of system dedifferentiation that was induced by alcohol administration (see Figure 3). We compared the dynamics of HRV after drinking an alcoholic beverage with the dynamics of HRV after drinking a non-alcoholic beverage. As mentioned above (see Introduction), alcohol administration decreases the activity of neurons that are specialized in relation to later formed behaviour and it does not significantly affect the activity of neurons specialized in relation to earlier formed behaviour [32,35,36]. Therefore, behaviour becomes less differentiated and detailed after alcohol administration.

Participants (N = 25, 5 females, 23 to 35 years old, mean = 26.82, median = 28) took part in the experiment twice, once in an alcohol condition and once in a control condition, with the time interval of 1–2 months. The order of the alcohol and control conditions was counterbalanced. In the beginning of each experiment, the participants were weighed and given a glass of beverage, which they were asked to drink within 30 min while watching an emotionally neutral video (BBC, “Planet Earth”). An alcoholic drink was given in the experimental condition and a non-alcoholic drink was given in the control condition.

The dose of alcohol was 1 g of ethanol (medical ethanol, 96%) to each 1 kg of a participant’s weight. A measured amount of alcohol was mixed with apple juice to achieve an overall drink volume of 750 mL. In the control condition, the participants drank apple juice mixed with water in the same proportion as in the alcohol condition. We measured breath alcohol content (BrAC, mg/L) using AlcoDigital AL7000 Pro Breathalyzer in the beginning and in the end of the experiment. BrAC was always equal to zero in the control condition. In the alcohol condition, the average level of BrAC before consuming an alcoholic drink was equal to zero and at the end of the experiment BrAC was equal to 0.72 ± 0.11 mg/L.

Heart rate was recorded during all experimental sessions. The SampEn, av-RR, and SDNN were calculated for sequential 5 min sections of RR-intervals during the drinking phase. The dynamics of sequential SampEn, av-RR, and SDNN values was compared in the alcohol and the control conditions using the Wilcoxon test.

#### 2.3.4. Experiment 4

**Figure 4:**
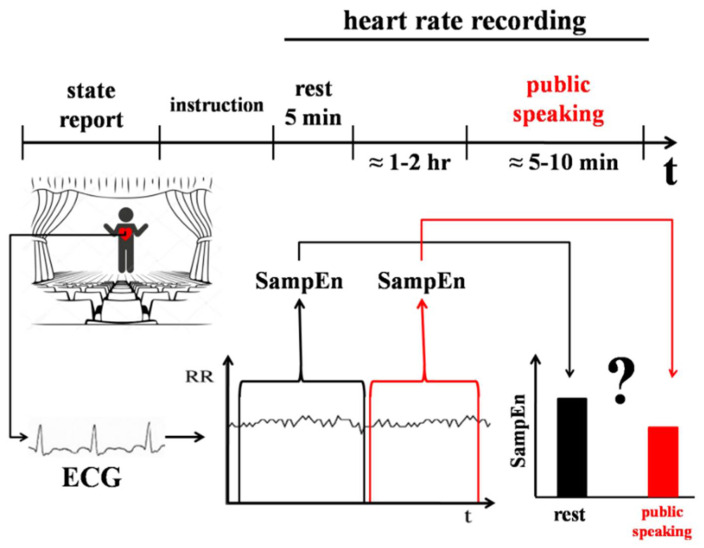
Design of Experiment 4. Public speaking was used as a model of social stress. Heart rate was recorded for 5 min at rest 1–2 h prior to public speaking and for the duration of public speaking which lasted 5–10 min. SampEn, av-RR, and SDNN were calculated for RR-intervals at rest and the period of stress and were compared between the conditions.

The aim of Experiment 4 was to study the dynamics of HRV in the condition of system dedifferentiation induced by stress (see Figure 4). The state of stress is usually characterized by high emotional arousal, reduced attention to details [37], preference to the most familiar behavioural strategies and habits [38], as well as making decisions that are typical for earlier stages of development [39]. Under stress, individuals tend to choose intuitive explanations, rather than rational [40]. From the neurobiological perspective, the biochemical diversity in the brain comes to three basic cascades during stress [41]. Overall, stress can be described as a state of temporal system dedifferentiation, which arises as a bias to earlier ways of adaptation [42].

Public speaking is part of the Trier Social Stress Test [43], which is widely used to model social stress. Participants (N = 13, 7 females, 21 to 30 years old, mean = 24.14, median = 24) were students of the Nizhny Novgorod State University, named after N.I. Lobachevski, who were taking part in academic conferences with oral reports.

The participants’ heart rate was recorded when they spoke in public (5–10 min) and while they rested (5 min) 1 or 2 h before their speech. SampEn, av-RR, and SDNN were calculated for the rest and stress sections of RR-intervals and were compared between the conditions using Wilcoxon test.

#### 2.3.5. Experiment 5

The aim of Experiment 5 was to analyze the dynamics of HRV during the process of learning. The beginning of learning a new task usually involves novelty and mismatch between the existing individual experience and current organism-environment relations. The same is observed when an individual experiences stress or intense emotions [44]. Moreover, a similar pattern of hormonal changes, i.e., an increase in cortisol levels and concentration of endogenous opioids, is observed during periods of learning, stress, and experiencing emotions [45]. Thus, we viewed the beginning stages of learning as a period of system dedifferentiation, which could be manifested in specific changes of HRV.

Participants (N = 35, 14 females, 18 to 38 years old, mean = 25.4, median = 25) had to learn to play a computer game. The task was to figure out the rules of the game. Before participants started to play, they were briefly familiarized with the apparatus. Then, they read an instruction, which translates as follows: “You are invited to play a computer game. The game has several levels and your general aim is to pass as many levels as you can. You don’t know the rules so you have to figure them out by yourself. All events of the game will take place on the screen and the only thing you will need is a computer mouse. You have unlimited time. You can stop playing if you feel bored or get tired. If you are ready to start, click “Play””. Participants played the game until they felt that they wanted to stop or that they achieved the end of the game by passing the last level.

The computer game had been developed using “A-Ware” software (Sozinov A.A., Bokhan A.I.) and was used previously to study behavioural characteristics and strategies in situations of unpredictability, novelty, and mismatching during forming new experience [46]. The design of the game involved a target object that was hidden within a playing field on the screen. The task was to find the target object and to draw a rectangle around it with the cursor as closely to its borders as possible. Good game performance was rewarded with points and players could see their points on the screen after each trial. The maximum possible number of points was 10,000. Once a player achieved 10,000 points in three sequential trials, she/he progressed to the next level. The game consisted of eight levels. The number of target objects (or their size) increased by one with each level passed.

**Figure 5:**
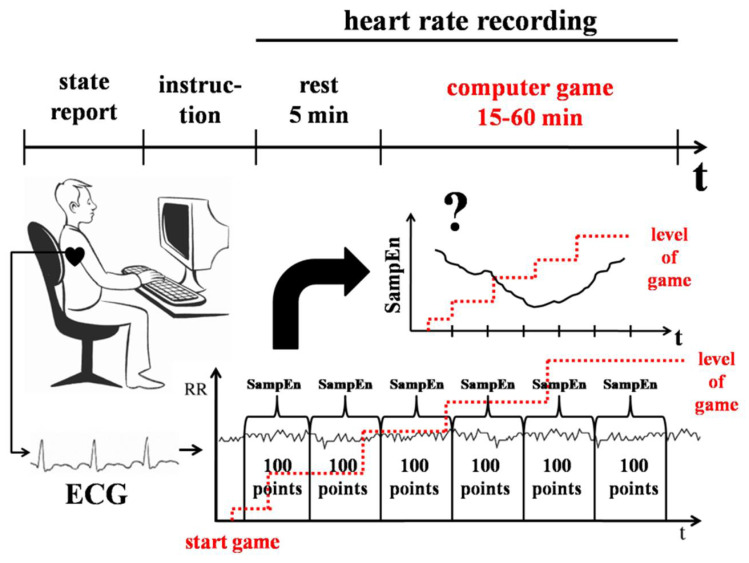
Design of Experiment 5. Heart rate was recorded while participants were learning to play a computer game. SampEn, av-RR, and SDNN were calculated for sequential 100 points sections of RR-intervals from the start of the game. The average dynamics of SampEn, av-RR, and SDNN values during the game were analyzed.

The participant’s heart rate was recorded during the game (Figure 5). SampEn, av-RR, and SDNN values were calculated for sequential RR-intervals with a running window (window length—was 100 points, the shift of running—10 points) from the start to the end of the game. To control for the factor of motivation, participants were given an opportunity to stop the game at any time. Therefore, the number of levels that participants passed was different (median = 3). Thus, we analyzed the dynamics of SampEn, av-RR, and SDNN at the beginning (about 150 s from the start) of the first level, which was passed by 26 participants. The heart rate indexes for the first section of RR-intervals from the start of the game were compared with following sections, using the Wilcoxon test.

### 2.4. Data Analyses

Statistical analyses were performed using STATISTICA10 software (http://documentation.statsoft.com/; STA999K347150-W; Expires 21.12.2021). For data used in statistical analyses, see Supplementary Material.

Distributions of all the variables were tested for normality by Shapiro-Wilk’s test. We calculated the medians and interquartile ranges and used nonparametric tests (Wilcoxon test and Mann-Whitney test) to compare distributions different from the normal distribution. In other cases, we calculated mean values and standard errors, and used parametric tests (*t*-test) to compare distributions. To evaluate the dynamics of variables we used Friedman test. To check the correlations between variables, we used Spearman’s rank correlation coefficient. Significance was assumed when *p* < 0.05.

## 3. Results

### 3.1. Dynamics of HRV During Performing a Linguistic Task Using Native and Foreign Languages (Experiment 1)

We compared SampEn, SDNN and av-RR values during the periods of performing the linguistic task in native (Russian) and foreign (German) languages in two groups of participants: those who had been learning German for 1–5 years, and those who had been learning it for more than five years. For descriptive statistics and results, see Table 1.

**Figure 6:**
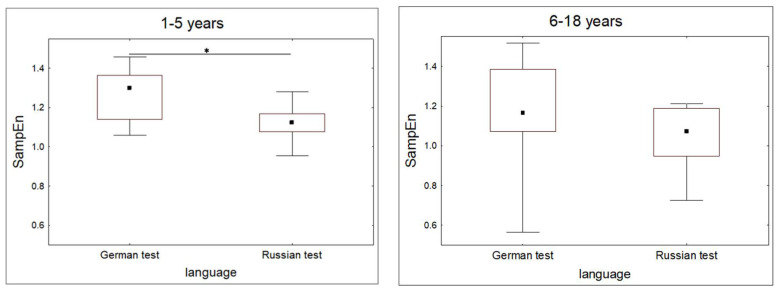
Median values (quartiles and range) of SampEn compared between the periods of performing the foreign (German) and native (Russian) language tasks by participants who had been learning the foreign language (German) for 1–5 years (**left** graph) and 6–18 years (**right** graph). * *p* < 0.01, Wilcoxon test.

In the group of participants who had been studying German for 1–5 years, SampEn was significantly lower when using Russian language as compared to using German language (Figure 6). Av-RR and SDNN did not differ between the two conditions in this group. In the group of participants who had been studying German for 6–18 years, SampEn, av-RR, and SDNN did not differ between the two conditions.

The analysis of behavioral performance and response time (ms) showed that when the participants of both groups were using foreign language, they made more mistakes and responded slower than when they were using native language (Table 2).

These results show that irregularity of the heart rate is lower when participants perform a task in their native language than when they perform the same task in a foreign language, if they have been learning it for a comparatively short period of time (1–5 years). Irregularity of the heart rate did not differ between the conditions when native and foreign languages were used in participants who had been learning the foreign language for a considerable period of time (6–18 years). It is important to note that irregularity of the heart rate during behaviour, but not its frequency or variability, was associated with the age when this behaviour had been formed.

### 3.2. Dynamics of HRV When Using Mathematical and General Vocabulary (Experiment 2)

**Figure 7:**
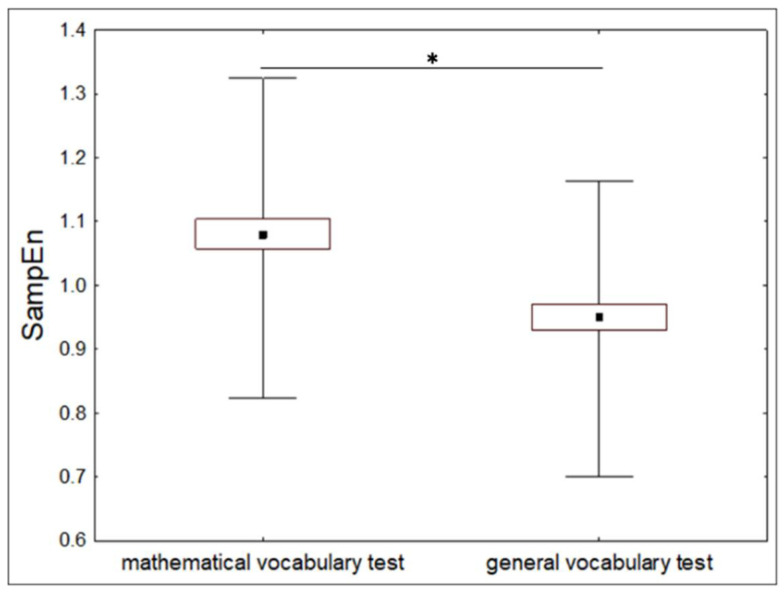
Mean values (±standard errors; range) of SampEn as compared between the intervals of performing a task using mathematical vocabulary and everyday vocabulary. * *p* < 0.01, *t*-test.

We compared SampEn, SDNN, and av-RR values during the periods when the participants were using mathematical and general vocabulary. For descriptive statistics and the results of comparisons, see Table 3. SampEn was significantly lower during performing the task involving general vocabulary than the task involving mathematical vocabulary (Figure 7). Av-RR and SDNN did not differ significantly between the two tasks.

The participants made more mistakes when using mathematical vocabulary (median = 6; Q1 = 4; Q3 = 8) as compared to general vocabulary (median = 2; Q1 = 2; Q3 = 3) (Wilcoxon test T = 0, Z = 4.7, *p* < 0.001). Response times were longer (Wilcoxon test T = 21, Z = 4.73, *p* < 0.001) when using mathematical vocabulary (median = 4488 ms; Q1 = 3254 ms; Q3 = 6502 ms) than general vocabulary (median = 3902 ms; Q1 = 2779 ms; Q3 = 5336 ms).

No significant correlation was observed between the HRV indexes and the behavioural characteristics (see Table 4). Thus, the differences in SampEn between the tasks could not be accounted for by the differences in performance.

These results indicate that the irregularity of the heart rate is lower when individuals are using knowledge acquired earlier in their development than knowledge acquired later, i.e., heart rate irregularity is associated with the age the current behaviour was formed. We did not observe such association for the frequency or variability of the heart rate.

### 3.3. Dynamics of HRV During Alcohol Administration (Experiment 3)

We tested whether the dynamics of heart rate indexes during alcohol administration consisted of non-random changes. Values of SampEn, SDNN, and av-RR for each 5-min section of RR-intervals were compared pair-wisely in the alcohol and control conditions. The results of these comparisons are shown in Table 5, Table 6 and Table 7.

**Figure 8:**
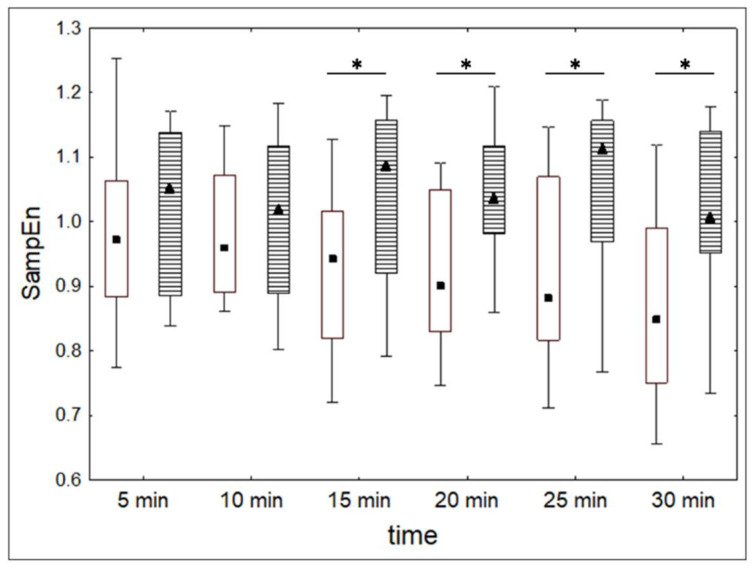
Median values (quartiles and range) of SampEn for consecutive 5 min sections of the heart rate during the alcohol (square points) and control (triangular points) conditions. *—Wilcoxon pair-wise comparisons, *p* < 0.05.

SampEn values decreased significantly during the 30 min of drinking in the alcohol condition, while there was no registered change in the dynamics of SampEn in the control condition (Figure 8). In general, the SampEn values were lower in the alcohol condition as compared to the control condition at 15, 20, 25, and 30 min periods after the beginning of the experiment.

In the alcohol condition, av-RR increased during the period between 5 and 10 min after the beginning of drinking, followed by a significant decrease at the end of the experiment. In the control condition, av-RR significantly increased during drinking throughout the experiment. In general, av-RR was significantly lower in the alcohol condition as compared to the control condition.

No non-random changes were observed in the dynamics of SDNN values in either alcohol or control conditions. SDNN was significantly lower in the alcohol condition as compared to the control condition at 10, 20, 25, and 30 min periods after the beginning of the experiment.

All in all, the results have shown that alcohol administration, as a factor inducing the system dedifferentiation, reduced HRV and irregularity of the heart rate while increasing frequency of the heart rate. The dynamics of SampEn during alcohol administration was more consequent and linear than the dynamics of SDNN.

### 3.4. Dynamics of HRV During Public Speaking (Experiment 4)

**Figure 9:**
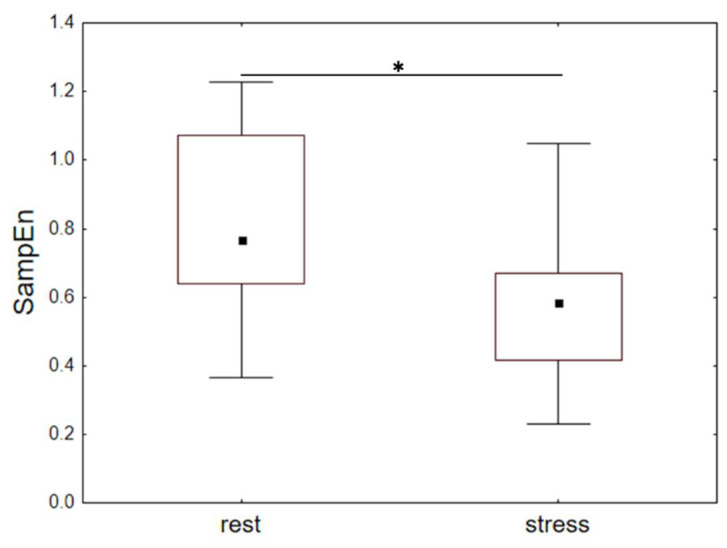
Median values (quartiles and range) of SampEn at rest and during social stress. * *p* < 0.05, Wilcoxon test.

We compared SampEn, SDNN, and av-RR values between the periods of rest (1–2 h before public speaking) and stress (during public speaking). The results are summarized in Table 8. SampEn was significantly lower during public speaking than at rest (Figure 9). The social stress condition also involved a decrease of SDNN and av-RR. Thus, social stress, as a factor inducing the system dedifferentiation, was accompanied by reduced irregularity of the heart rate, which was also observed in the condition of alcohol administration.

### 3.5. Dynamics of HRV During Learning to Play a Computer Game (Experiment 5)

We analyzed the dynamics of SampEn, av-RR, and SDNN at the beginning of the first level of the game. The heart rate indexes were calculated for the initial 100 RR-intervals followed by five subsequent sections of RR-intervals with a step in 10 points from the beginning of the game: 1–100, 10–110, 20–120, 30–130, 40–140, and 50–150 points. The indexes were compared between the first section (1–100 points) and five subsequent sections (10–110, 20–120, 30–130, 40–140, and 50–150 points). For descriptive statistics, see Table 9.

**Figure 10:**
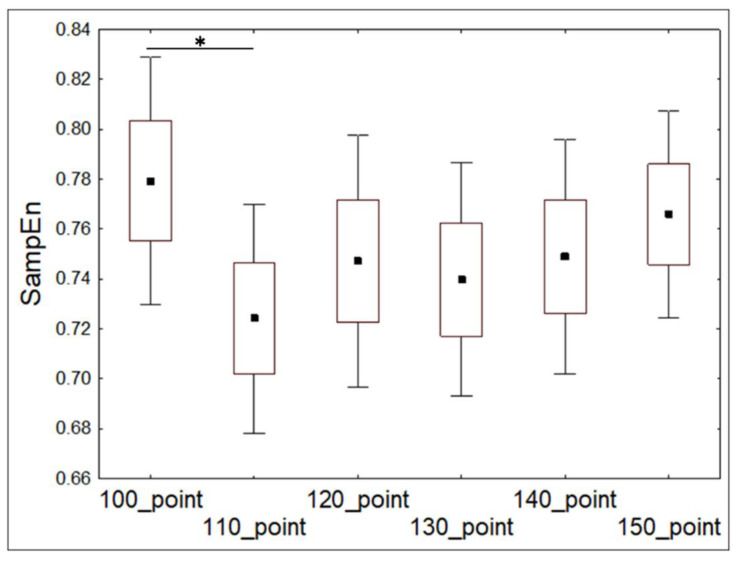
Mean values (±standard errors; range) of SampEn calculated for the initial 150 points of playing a computer game. *—*p* < 0.05, *t*-test for dependent samples.

SampEn calculated for the section of 1–100 points was significantly higher than the SampEn calculated for the section of 10–110 points (t = 2.42, *p* < 0.05, *t*-test dependent samples) (Figure 10). No other significant difference in SampEn was observed between the sections of RR-intervals during the initial 150 points of learning to play the game. Av-RR that was calculated for the section of 1–100 points was significantly lower than 10–110 points (t = −2.11, *p* < 0.05, *t*-test dependent samples). No other significant difference in av-RR was observed between the sections of RR-intervals during the initial 150 points of learning to play the game. SDNN for the section of 1–100 points was significantly higher than sections of 10–110 (t = 3.46, *p* < 0.01), 20–120 (t = 3.53, *p* < 0.01), 30–130 (t = 3.11, *p* < 0.01), 40–140 (t = 3.01, *p* < 0.01), and 50–150 (t = 2.74, *p* < 0.05, *t*-test dependent samples) points from the beginning of learning to play the game.

These results showed that irregularity of the heart rate as well as its frequency decreased at the beginning of learning. Variability of the heart rate also decreased at the beginning of learning and was lower than at any further point of leaning analyzed in our study.

## 4. Discussion

The aim of the current study was to explore how the system organization of behaviour can be reflected in the irregularity of the heart rate, as measured by SampEn. In experiments reported in this paper, we show that behaviour based on early-formed experience, related to native language as opposed to foreign language, and general vocabulary as opposed to abstract mathematical vocabulary, is associated with a lower irregularity of the heart rate. From the positions of system-evolutionary theory, this can be viewed as a result of increasing of system differentiation in the organization of later formed behaviour. Behaviour that is formed at later stages of individual development is based on a larger set of functional systems that are represented in the brain by neuronal groups specialized in relation to different acts comprising this behaviour, each formed at a certain point of an organism’s life [2,17,20,47]. Therefore, the later formed behaviour is considered as more complex and detailed. Our results show that the regularity of the physiological processes underlying such behaviour decreases and their dynamics become more complex.

Heart rate irregularity, as a measure of non-linear dynamics of functional systems that are involved in the organization of current behaviour, is higher when this behaviour was formed later in individual development, but such differences were not observed for frequency of the heart rate, which implies that the higher heart rate irregularity cannot be explained by differences in the intensity of cognitive load engaging more internal recourses when performing later-formed behaviors. Therefore, we argue that this effect is related to characteristics of the functional systems that are actualized in the current behaviour.

It was shown that later-formed behaviour is subserved by the activity of a higher quantity of neural networks [20,21,47]. Therefore, we assume that when an individual is performing a later formed behaviour, his heart is involved in a more complex non-linear activity in order to achieve an adaptive coordination with the activity of neuronal elements of functional systems. This results in a decreased regularity of the heart rate.

We also show that irregularity of the heart rate is decreased during stress, alcohol administration, and at the beginning of learning. From the positions of system-evolutionary theory, this can be viewed as a result of system dedifferentiation in the organization of behaviour. In the context of system dedifferentiation, the number of actualized functional systems declines as the organization of behaviour becomes less complex [42], which is reflected in the reduced irregularity of the dynamics of physiological processes, as shown in our study.

The decrease in heart rate irregularity during public speaking shown in our study is in line with similar results obtained for examination stress [48] and hemorrhagic shock [49]. A state of shock can be considered as a higher degree of stress; heart rate irregularity that is observed in the state of shock is four times lower than heart rate irregularity during stress. This suggests that a decrease in heart rate irregularity may be quantitatively related to the degree of stress and system dedifferentiation induced by the stress.

We have not found any other studies that would show the dynamics of heart rate irregularity during alcohol administration, yet a decrease in heart rate variability, as measured by SDNN and HF (power of the spectrum of heart rate in the high-frequency range), during alcohol administration had been demonstrated previously [28].

A decrease of heart rate irregularity that was observed during the 30 min of drinking in the alcohol condition comports with the dynamics of ethanol absorption for the initial 20–40 min of alcohol intake reported in other studies [50]. At this initial stage of absorption, concentration of ethanol in the bloodstream grows before it gradually transitions to the next stage of ethanol elimination, when the concentration of ethanol in the bloodstream starts reducing. It is reasonable to assume that in a case of a longer experiment with alcohol administration (about 3 h), one can expect a gradual increase in heart rate irregularity, which would be slower than the initial decrease, as the stage of elimination lasts longer than the stage of absorption. It is worth noting that a similar decrease of heart rate irregularity, as measured by approximate entropy (ApEn), has been shown in experiments with cocaine administration [51], which can be viewed as a possible factor of the system dedifferentiation. Moreover, the cocaine-induced decrease in heart rate irregularity was dose-dependent. Therefore, we assume that decreasing heart rate irregularity followed after alcohol administration reflects and may be quantitatively related to the processes of system dedifferentiation.

The dynamics of heart rate irregularity at the beginning of learning can be viewed along with data on the pattern of hormonal changes, i.e., an increase in cortisol levels and concentration of endogenous opioids [45], typical for novel situations involving a mismatch between the existing individual experience and current organism-environment relations. The initial stages of learning can be described as a state of high emotional arousal, accompanied by a reduction in the actualization of later-formed systems, when an individual goes back to the states of earlier developmental stages [23], which could be viewed as an adaptive mechanism. Experiments with mathematical modelling have shown that without such a reduction in differentiation of the system organization of behaviour at the beginning of learning, it took animates a significantly longer time to find ways of solving a new task [42]. Thus, a decrease of heart rate irregularity during the initial stages of learning can also be viewed as a phenomenon that is associated with the processes of system dedifferentiation.

In addition, this view is supported by the results that were obtained in the studies of heart rate irregularity and emotional arousal. G. Valenza and coauthors [52] compared heart rate indexes during viewing the IAPS pictures, which elicited different levels of emotional arousal. They showed that the heart rate irregularity, as measured by ApEn, was lower when participants were viewing the pictures with a non-zero level of arousal than emotionally neutral pictures. I. Grossman and coauthors found a positive correlation between heart rate variability and the number of decisions based on wise reasoning about political and social problems [53].

It is important to note that we have not found any significant changes in the dynamics of av-RR or SDNN when comparing them between behaviors that are formed at earlier and later stages of individual development (Experiment 1 and 2). These results imply that different modes of heart activity manifested in the dynamics of SampEn cannot be explained by the difference in intensity of cognitive load, which demands unequal amounts of internal resources, cognitive control, and efforts when performing early- and later-formed behaviors. Typical decreases of av-RR and SDNN (Experiment 3 and 4) were observed during stress and alcohol administration, which had been reported in many previous studies. However, we believe that decreasing SampEn in these situations can also be explained by the dynamics of functional systems. For example, in Experiment 5 we observed decreasing SampEn and SDNN, accompanied by increasing av-RR. Thus, decreasing SampEn is not always observed when av-RR and SDNN are also decreasing. This suggests that SampEn reflects not only the basic states of general well-being of the organism, but can also be used to study the system processes orchestrating behaviour.

In summary, we argue that heart rate variability originates from complex system processes subserving behaviour, and it would be inaccurate to view it simply as a result of excitatory and inhibitory commands from the structures of the central nervous system. In the framework of system-evolutionary theory, behaviour is considered as an individual’s activity at the whole-organism level. Therefore, changes in activity of the heart are viewed as a result of its coordination with other elements of functional systems actualized in the current behaviour, particularly with changes in the sets of activated neurons distributed across the cerebral cortex and sub-cortical structures and specialized in relation to this behaviour. This coordination is subserved via a number of afferent and efferent nerve fibers of the parasympathetic and sympathetic autonomic nervous system, as well as neurons of the intracardiac nervous system tightly-distributed in the myocardium [54].

**Figure 11:**
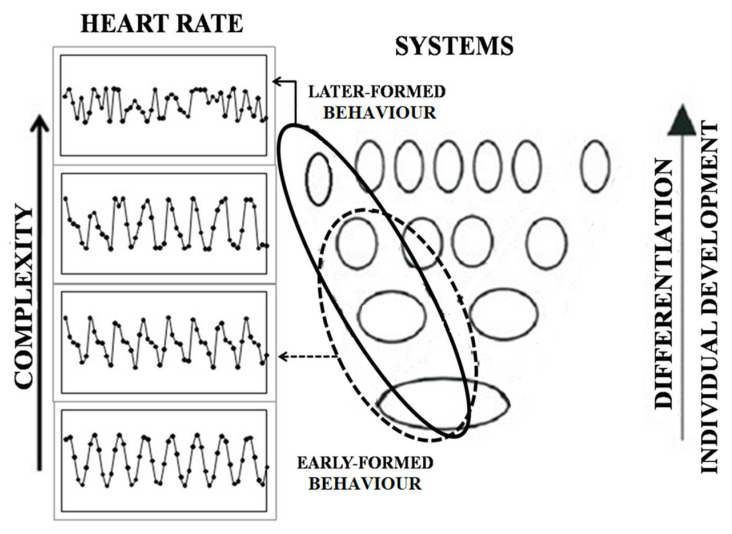
Heart rate irregularity and the number of functional systems actualized in current behaviour. The ovals depict functional systems formed at different stages of individual development. The selected groups of ovals illustrate combinations of functional systems that provide realization of earlier (dotted line) and later (solid line) formed behaviour. Heart rate irregularity, as measured by SampEn, increases along with behavioural differentiation in the process of individual development.

The results of this study allow for us to assume a positive correlation between the number of functional systems and inter-system connections subserving current behaviour, which become more complex in individual development, and heart rate irregularity observed during this behaviour (Figure 11). The more functional systems actualized in the behaviour, the more elements the heart coordinates its activity with in order to achieve a common whole-organism outcome, which is reflected in higher heart rate irregularity.

Integrating results of the five experiments, the study proposes that SampEn of the heart rate can be used as a new tool to study psychological processes and organization of behaviour. Many previous studies had demonstrated the sensitivity of SampEn to physiological and behavioural dynamics, which can be applied for diagnostics of optimal and extremal functional states [28,55], as well as various clinical pathologies [28,56]. Our study has demonstrated the potential of using SampEn in psychological research in order to study the system organization of behaviour.

An important output of this study is that the dynamics of functional systems supporting current behavior is reflected not only in activity of the brain, but also in the activity of the rest of the body. Functional systems subserving behaviour are not entirely neuronal systems but they also consist of elements located in different parts of the body. All elements of functional systems, neuronal and non-neuronal, continuously change their activity in order to achieve an effective cooperation resulting in an adaptive outcome relevant for the whole organism.

## 5. Conclusions

Heart rate irregularity is higher when an individual performs behaviour formed at later stages of his development (e.g., using foreign language vs. native language and later acquired words vs. earlier acquired words). The processes of system dedifferentiation (a reduction of the number of functional systems subserving current behaviour) that were observed during stress, after alcohol administration, and at the beginning stages of learning are accompanied by a decrease in heart rate irregularity. Thus, irregularity of the heart rate reflects characteristics of the system organization of behaviour.

## Figures and Tables

**Table 1:** Heart rate indexes during performing a language task in native (Russian) and foreign (German) languages for groups of participants who had been learning the foreign language (German) for 1–5 years and 6–18 years ^1^.

HR Index	Group	Language	Med	Q1	Q3	Comparison
SampEn	1–5 years	German	1.30	1.14	1.36	T = 7, Z = 3.42, *p* < 0.01
		Russian	1.12	1.08	1.17	
	6–18 years	German	1.17	1.07	1.38	T = 16, Z = 1.51, *p* = 0.13
		Russian	1.07	0.95	1.19	
av-RR (ms)	1–5 years	German	705	647	759	T = 78, Z = 0.33, *p* = 0.74
		Russian	738	641	765	
	6–18 years	German	713	584	749	T = 20, Z = 1.16, *p* = 0.25
		Russian	695	579	756	
SDNN (ms)	1–5 years	German	49.39	40.12	57.50	T = 79, Z = 0.28, *p* = 0.78
		Russian	48.81	42.25	62.55	
	6–18 years	German	48.05	37.20	52.97	T = 25, Z = 0.71, *p* = 0.48
		Russian	47.97	33.91	57.92	

^1^ Median values (Med) and quartiles (Q1 and Q3) are presented along with the results of Wilcoxon test comparisons between the periods of using native and foreign languages.

**Table 2:** Task performance in native (Russian) and foreign (German) languages for groups of participants who had been learning the foreign language (German) for 1–5 years and 6–18 years ^1^.

Variables	Group	Language	Med	Q1	Q3	Comparison
number of mistakes	1–5 years	German	12	6	13	T = 0, Z = 3.62, *p* < 0.01
		Russian	0	0	0	
	6–18 years	German	5	2	10	T = 0, Z = 2.93, *p* < 0.01
		Russian	0	0	0	
response time (ms)	1–5 years	German	8166	6615	10,953	T = 0, Z = 3.62, *p* < 0.01
		Russian	4271	3787	5191	
	6–18 years	German	6803	3565	8412	T = 4, Z = 2.58, *p* < 0.01
		Russian	3300	2579	3835	

^1^ Median values (Med) and quartiles (Q1 and Q3) are presented along with the results of Wilcoxon test comparisons between the periods of using native and foreign languages.

**Table 3:** Heart rate indexes during performing a task using mathematical and everyday vocabulary ^1^.

HR Index	Test	Med	Q1	Q3	Comparison
SampEn	mathematical terms	1.09	0.98	1.15	t = 7.70, *p* < 0.01, *t*-test
	general vocabulary	0.97	0.86	1.04	
av-RR (ms)	mathematical terms	803	739	916	t = −1.79, *p* = 0.08, *t*-test
	general vocabulary	814	754	922	
SDNN (ms)	mathematical terms	57.30	45.41	76.68	T = 309, Z = 0.09, *p* = 0.92, Wilcoxon test
	general vocabulary	56.66	44.52	78.32	

^1^ Median values (Med) and quartiles (Q1 and Q3) are presented along with the results of Wilcoxon and *t*-test pair-wise comparisons between the periods of using mathematical and everyday vocabulary.

**Table 4:** Spearman correlation coefficients between the heart rate indexes and behavioural characteristics when using mathematical and general vocabulary.

**Mathematical Vocabulary**	**SampEn**	**av-RR (ms)**	**SDNN (ms)**
number of mistakes	0.07	0.06	−0.09
response time (ms)	−0.27	0.13	−0.32
**General vocabulary**	**SampEn**	**av-RR (ms)**	**SDNN (ms)**
number of mistakes	0.09	0.16	0.01
response time (ms)	0.11	−0.28	0.12

**Table 5:** SampEn values for consecutive 5 min sections of the heart rate during the alcohol and control conditions ^1^.

Statistics	Conditions	Time Period after the Start of Drinking						Friedman Test
		5 min	10 min	15 min	20 min	25 min	30 min	
Med	alcohol	0.97	0.96	0.94	0.90	0.88	0.85	X^2^ = 13.62, *p* < 0.03
	control	1.07	1.02	1.09	1.04	1.11	1.04	X^2^ = 1.60, *p* = 0.90
Q1	alcohol	0.88	0.89	0.82	0.83	0.82	0.75	
	control	0.89	0.89	0.92	0.98	0.97	0.95	
Q3	alcohol	1.06	1.07	1.02	1.05	1.07	0.99	
	control	1.14	1.13	1.18	1.12	1.18	1.15	
	Wilcoxon test	T = 121 Z = 1.12 *p* = 0.26	T = 127 Z = 0.96 *p* = 0.34	T = 71 Z = 2.46 *p* < 0.05	T = 46 Z = 3.14 *p* < 0.01	T = 79 Z = 2.25 *p* < 0.05	T = 73 Z = 2.41 *p* < 0.05	

^1^ Median values (Med) and quartiles (Q1 and Q3) are shown along with the results of Friedman test and Wilcoxon pair-wise comparisons between the alcohol and control conditions.

**Table 6:** Av-RR values (ms) for consecutive 5 min sections of the heart rate during the alcohol and control conditions ^1^.

Statistics	Conditions	Time Period after the Start of Drinking						Friedman Test
		5 min	10 min	15 min	20 min	25 min	30 min	
Med	alcohol	716	734	729	718	717	715	X^2^ = 23.52, *p* < 0.01
	control	770	796	804	829	832	811	X^2^ = 40.63, *p* < 0.01
Q1	alcohol	680	676	649	659	657	632	
	control	697	736	745	752	739	748	
Q3	alcohol	782	797	785	792	794	764	
	control	870	921	898	903	940	916	
	Wilcoxon test	T = 87 Z = 2.25 *p* < 0.05	T = 57 Z = 3.01 *p* < 0.01	T = 37 Z = 3.52 *p* < 0.01	T = 16 Z = 4.05 *p* < 0.01	T = 25 Z = 3.82 *p* < 0.01	T = 24 Z = 3.85 *p* < 0.01	

^1^ Median values (Med) and quartiles (Q1 and Q3) are shown along with the results of Friedman test and Wilcoxon pair-wise comparisons between the alcohol and control conditions.

**Table 7:** SDNN values (ms) for consecutive 5 min sections of the heart rate during the alcohol and control conditions ^1^.

Statistics	Conditions	Time Period after the Start of Drinking						Friedman Test
		5 min	10 min	15 min	20 min	25 min	30 min	
Med	alcohol	70.99	67.78	70.26	69.99	67.34	65.42	X^2^ = 8.90, *p* = 0.11
	control	68.99	80.66	73.62	78.15	78.09	74.90	X^2^ = 4.69, *p* = 0.45
Q1	alcohol	54.32	52.34	46.61	46.03	48.47	49.35	
	control	54.55	65.01	59.90	58.90	69.42	58.87	
Q3	alcohol	80.97	76.46	76.33	80.93	76.20	70.47	
	control	100.77	92.83	97.11	108.10	96.74	120.74	
	Wilcoxon test	T = 136 Z = 1.01 *p* = 0.32	T = 68 Z = 2.73 *p* < 0.01	T = 111 Z = 1.64 *p* = 0.101	T = 62 Z = 2.88 *p* < 0.01	T = 64 Z = 2.83 *p* < 0.01	T = 59 Z = 2.96 *p* < 0.01	

^1^ Median values (Med) and quartiles (Q1 and Q3) are shown along with the results of Friedman test and Wilcoxon pair-wise comparisons between the alcohol and control conditions.

**Table 8:** Heart rate indexes at rest and during social stress ^1^.

HR Index	Period	Med	Q1	Q3	Wilcoxon Test
SampEn	rest	0.77	0.64	1.07	T = 16, Z = 2.06, *p* < 0.05
	stress	0.58	0.42	0.67	
av-RR (ms)	rest	585	505	686	T = 8, Z = 2.62, *p* < 0.05
	stress	470	411	531	
SDNN (ms)	rest	56.14	47.99	69.28	T = 0, Z = 3.18, *p* < 0.01
	stress	43.26	27.19	54.11	

^1^ Median values (Med) and quartiles (Q1 and Q3) are presented along with the results of Wilcoxon pair-wise comparisons between the periods of rest and social stress during speaking in public.

**Table 9:** Heart rate indexes (means and standard errors) calculated for the initial 150 points of playing a computer game.

HR Index	Point of Time from the Start of Playing the Game					
	100	110	120	130	140	150
SampEn	0.78 ± 0.02	0.73 ± 0.02	0.75 ± 0.02	0.74 ± 0.02	0.75 ± 0.02	0.76 ± 0.02
av-RR (ms)	789 ± 19.02	793 ± 19.73	792 ± 20.12	792 ± 20.24	791 ± 20.21	791 ± 20.06
SDNN (ms)	58.91 ± 4.66	52.19 ± 4.01	50.79 ± 3.84	50.04 ± 3.71	49.88 ± 3.81	49.93 ± 3.52

## Supplementary Materials

The supplementary materials are available online at http://www.mdpi.com/1099-4300/20/6/449/s1.
